# Joint Effects of Colorectal Cancer Susceptibility Loci, Circulating 25-Hydroxyvitamin D and Risk of Colorectal Cancer

**DOI:** 10.1371/journal.pone.0092212

**Published:** 2014-03-26

**Authors:** Linda T. Hiraki, Amit D. Joshi, Kimmie Ng, Charles S. Fuchs, Jing Ma, Aditi Hazra, Ulrike Peters, Elizabeth W. Karlson, Edward Giovannucci, Peter Kraft, Andrew T. Chan

**Affiliations:** 1 Program in Molecular and Genetic Epidemiology, Department of Epidemiology, Harvard School of Public Health, Boston, Massachusetts, United States of America; 2 Medical Oncology, Dana Farber Cancer Institute, Boston, Massachusetts, United States of America; 3 Channing Division of Network Medicine, Department of Medicine, Brigham and Women's Hospital and Harvard Medical School, Boston, Massachusetts, United States of America; 4 Public Health Sciences Division, Fred Hutchinson Cancer Research Center, Seattle, Washington, United States of America; 5 Division of Rheumatology, Department of Medicine, Brigham and Women's Hospital and Harvard Medical School, Boston, Massachusetts, United States of America; 6 Departments of Epidemiology and Nutrition, Harvard School of Public Health, Boston, Massachusetts, United States of America; 7 Division of Gastroenterology, Department of Medicine, Massachusetts General Hospital, Boston, Massachusetts, United States of America; MOE Key Laboratory of Environment and Health, School of Public Health, Tongji Medical College, Huazhong University of Science and Technology, China

## Abstract

**Background:**

Genome wide association studies (GWAS) have identified several SNPs associated with colorectal cancer (CRC) susceptibility. Vitamin D is also inversely associated with CRC risk.

**Methods:**

We examined main and joint effects of previously GWAS identified genetic markers of CRC and plasma 25-hydroxyvitamin D (25(OH)D) on CRC risk in three prospective cohorts: the Nurses' Health Study (NHS), the Health Professionals Follow-up Study (HPFS), and the Physicians' Health Study (PHS). We included 1895 CRC cases and 2806 controls with genomic DNA. We calculated odds ratios and 95% confidence intervals for CRC associated with additive genetic risk scores (GRSs) comprised of all CRC SNPs and subsets of these SNPs based on proximity to regions of increased vitamin D receptor binding to vitamin D response elements (VDREs), based on published ChiP-seq data. Among a subset of subjects with additional prediagnostic 25(OH)D we tested multiplicative interactions between plasma 25(OH)D and GRS's. We used fixed effects models to meta-analyze the three cohorts.

**Results:**

The per allele multivariate OR was 1.12 (95% CI, 1.06–1.19) for GRS-proximalVDRE; and 1.10 (95% CI, 1.06–1.14) for GRS-nonproxVDRE. The lowest quartile of plasma 25(OH)D compared with the highest, had a multivariate OR of 0.63 (95% CI, 0.48–0.82) for CRC. We did not observe any significant interactions between any GRSs and plasma 25(OH)D.

**Conclusions:**

We did not observe evidence for the modification of genetic susceptibility for CRC according to vitamin D status, or evidence that the effect of common CRC risk alleles differed according to their proximity to putative VDR binding sites.

## Introduction

Both inherited and modifiable risk factors have been identified for colorectal cancer (CRC). To date, genome wide association studies (GWAS) have identified 32 SNPs at 23 independent loci associated with CRC [1]–[16]. A substantial body of evidence also demonstrates an inverse association between vitamin D status and CRC [17]–[24]. Nonetheless, data examining the potential interaction between genetic susceptibility to CRC and an environmental factor such as vitamin D status are lacking.

There are plausible mechanisms by which genetic susceptibility to CRC may vary according to vitamin D status. First, one of the primary modes of action of vitamin D is via influence of gene transcription by binding of the active form 1,25-dihydroxy-vitamin D (1,25(OH)_2_D_3_) to the nuclear vitamin D receptor (VDR) [25]. A prior study utilizing chromatin immunoprecipitation with massively parallel sequencing (ChIP-seq) to identify vitamin D receptor (VDR) protein– DNA binding interactions observed that VDR binding sites were significantly enriched near autoimmune and cancer associated genes [26], including 3 previously identified CRC-associated SNPs. Second, although our understanding of the functional implications of many CRC-associated SNPs is limited, it is possible that some of these loci may be associated mechanistically with pathways also influenced by vitamin D.

Thus, we examined the joint effects of genetic markers of CRC previously identified by GWAS and plasma 25(OH)D on CRC risk in three prospective cohorts: the Nurses' Health Study (NHS), the Health Professionals Follow-up Study (HPFS), and the Physicians' Health Study (PHS). We also specifically explored the possibility that vitamin D status may differentially influence risk of CRC according to genetic variants proximal to regions of VDR binding demonstrated in functional ChIP-seq studies.

## Methods

### Study Population

Our study included three case-control studies of CRC nested within the Nurses' Health Study (NHS), the Health Professionals Follow-up Study (HPFS) and the Physicians' Health Study (PHS). The NHS was established in 1976 when 121,700 US female registered nurses aged 30 to 55 years returned mailed questionnaires on risk factors for cancer and cardiovascular disease [27], [28]. The HPFS was established in 1986 when 51,529 male health professionals aged 40 to 75 years responded to a similar questionnaire [29]. In both cohorts, participants have returned questionnaires every 2 years to update information with response rates exceeding 90% [27], [28]. In 1989-90, 32,826 NHS participants and in 1993-95, 18,018 HPFS participants returned a blood specimen on ice packs. In 2001–04, 29,684 women in NHS and 13,956 men in HPFS who had not previously provided a blood specimen mailed in a ‘swish-and-spit’ sample of buccal cells. On receipt, blood and buccal cells were centrifuged, aliquoted, and stored at −70°C [30]. The Physicians' Health Study was a randomized, double-blind, placebo-controlled trial of aspirin and beta-carotene for the primary prevention of cancer and cardiovascular disease among 22,071 U.S. male physicians ages 40 to 84 years enrolled in 1982 [31]. Participants with a prior diagnosis of heart disease, cancer (except nonmelanoma skin cancer), renal or liver disease, peptic ulcer, or gout or used vitamin A or beta-carotene supplements, were excluded. Between 1982 and 1984, 14,916 men (more than 70% of participants) returned blood samples by mail which were divided into aliquots and stored at −82°C (later, at −140°C) [32]. This study was approved by the Human Subjects Committee at Brigham and Women's Hospital and the Harvard School of Public Health in Boston, MA, USA. All participants provided informed consent.

In all three cohorts, incident cases of CRC were identified by follow-up questionnaires and confirmed by medical records review or through mortality follow-up. In each cohort, up to 3 controls were randomly selected from those who were alive and free of cancer at the time of case ascertainment. In NHS and HPFS, controls were matched to each case on ethnicity, year of birth and month/year of blood or buccal sampling [30]; in PHS, controls were additionally matched on smoking status [32].

### Laboratory Assessment

We previously measured plasma levels of 25(OH)D through a radioimmunosorbent assay in the laboratory of Dr. Bruce W. Hollis (Medical University of South Carolina, Charleston, SC). The median intra-assay coefficient of variation from blinded quality-control samples was 11.8% in NHS, 10.1% in HPFS, and 13.8% in PHS. Cases and their controls were analyzed in the same batch, and laboratory personnel were blinded to case, control, and quality-control status [23], [32], [33].

### Genotyping

Genomic DNA was extracted from blood samples (HPFS, NHS, PHS) and buccal cells (NHS, HPFS) using conventional methods. We used the TaqMan Open Array SNP genotyping platform (Biotrove, Woburn, MA) with 384-well format TaqMan assays to genotype the following CRC-associated variants identified from previous GWAS: rs6691170, rs6687758, rs10936599, rs16892766, rs6983267, rs10795668, rs3802842, rs10505477, rs7014346, rs7136702, rs11169552, rs4444235, rs4779584, rs9929218, rs4939827, rs10411210, rs961253, rs4925386 [4], [8]-[10], [12], [15], [16]. TaqMan primers and probes were designed using Primer Express Oligo Design software v2.0 (ABI PRISM). Primers, probes, and conditions for genotyping assays are available upon request. We genotyped rs2151512 on Taqman as a surrogate for rs4925386 (linkage disequilibrium r^2^ 1.0 in the HapMap CEU population) since genotyping by Taqman for rs4925386 on chromosome 20q13.33 was not successful.A subset of participants (954 cases and 1328 controls) had blood genomic DNA successfully genotyped using Illumina HumanOmniExpress. Missing SNP data was imputed to HapMap II release 22 using MACH [34]. All genotyping underwent standard quality control including concordance checks for blinded and unblinded duplicates and examination of sample and SNP call rates. The call rate was >97% for all samples and >98% for all SNPs.

### Statistical Analysis

We included a total of 1895 CRC cases and 2806 controls with genotype information assembled from NHS, HPFS, and PHS for our GRS. Within each cohort, we calculated allelic odds ratios and 95% confidence intervals for CRC associated with each SNP and for genetic risk scores (GRSs). The GRS is an allelic scoring system incorporating each of the specified risk alleles associated with CRC based on prior GWAS to assign a single quantitative index of genetic risk to each subject. Our GRS assumes an additive allelic effect with carriage of an increasing number of copies of each risk variant.

We constructed a GRS comprised of 18 Taqman CRC susceptibility SNPs (GRS-18). We also examined GRSs comprised of a subset of these SNPs based upon the 10 SNPs examined in. a ChiP-seq analysis (GRS-10) conducted by Ramagopalan et al. In this analysis, 3 CRC-associated SNPs were proximal (within 150 kb on either side of main disease-associated SNP) to increased VDR binding (vitamin D response elements (VDRE)) (GRS-proximalVDRE), and 7 SNPs were not proximal to areas of increased VDR binding (GRS-nonproxVDRE) [26]. Among the 2,282 subset of individuals with additional GWAS data, we created a GRS comprised of 31 CRC associated SNPs from GWAS (GRS-31). We also calculated GRSs using the effect estimates and standard errors for these same SNPs from large scale GWAS [35] and tested for differences in the GRS-proximalVDRE and GRS-nonproxVDRE.

For analyses of the joint effect of plasma 25(OH)D and GRS-18, we included the 881 cases and 1566 controls in NHS, HPFS, and PHS who also had previously measured 25(OH)D prior to CRC diagnosis [23], [32]. This was repeated for the joint effects of GRS-31 and pre-CRC 25(OH)D levels among the 672 cases and 909 controls with both GWAS data and vitamin D levels. We calculated odds ratios and 95% confidence intervals for CRC associated with each 1 ng/mL increase in 25(OH)D; high versus low vitamin D according to a threshold level of 25(OH)D associated with lower CRC risk (≥32 ng/mL); and quartiles of 25(OH)D [32]. We tested for multiplicative interactions between GRSs and vitamin D using a product term in the model and assessing its significance by the Wald method.

We adjusted our genetic analyses for age at sample collection, race, cohort, and type of sample (blood or cheek). Analyses which also incorporated plasma 25(OH)D additionally adjusted for season of blood draw and 25(OH)D analysis batch. We also used multivariate models which included additional CRC risk factors, including regular aspirin use (yes or no), body mass index (BMI; in tertiles), physical activity (in tertiles), history of CRC in a parent or sibling (yes or no), smoking status (never, former or current smoker), alcohol consumption (0–4.9, 5–9.9, 10–14.9 or ≥ 15.0 g per day), and consumption of beef, pork or lamb as a main dish (0–3 times per month, once a week, 2–4 times per week or ≥5 times per week). In NHS and HPFS we also included regular non-steroidal anti-inflammatory drugs (NSAIDs) use (yes or no) and energy-adjusted calcium and folate intake (in tertiles). Study specific estimates were meta-analyzed to determine a combined OR and 95% CI using inverse variance weights [36]. Fixed effect model estimates were used as all tests of heterogeneity were non-significant (p>0.05).

## Results

Our analysis included 1895 CRC cases and 2806 controls assembled from the three cohorts. Table 1 summarizes the baseline characteristics of each cohort. The direction and magnitude of our individual SNP effect estimates were comparable to those of the original reports as well as those observed in the Genetics and Epidemiology of Colorectal Cancer Consortium (GECCO) and Colon Cancer Family Registry (CCFR). GECCO/CCFR encompasses 13 studies, including NHS, HPFS, and PHS with genetic information on a total of 10,061 cases and 12,768 controls (Table 2).

**Table 1:** Baseline characteristics of cases and controls in NHS, HPFS and PHS.

	NHS		HPFS		PHS	
	cases	controls	cases	controls	cases	controls
	n = 922	n = 1436	n = 586	n = 871	n = 387	n = 499
Age at diagnosis	66.8 (9.2)	66.0 (9.1)	69.2 (9.2)	70.0 (9.1)	68.9 (9.1)	68.2 (9.4)
Age at sample draw (year, mean)	65.4 (8.8)	63.2 (8.5)	64.8 (8.5)	64.9 (8.4)	59.3 (9.0)	57.4 (8.6)
Mean 25(OH)D ng/mL	24.5 (9.8)	26.5 (9.7)	28.3 (9.3)	29.3 (9.4)	25.7 (9.7)	25.2 (8.9)
Non-white (%)	1.4	0.4	8	7	4	5
BMI (mean(SD))	26.1 (5.1)	26.0 (5.0)	26.2 (3.3)	25.6 (3.3)	25.2 (2.8)	24.6 (2.6)
Former or current smoker (%)	58	55	54	51	59	57
Alcohol consumption ≥15 g or ≥2 drinks/day	14	13	32	30	30	27
Beef, pork or lamb as a main dish ≥1/day (%)	16	14	14	14	3	4
Physical activity ≤7.6 METs-h/week or ≤3 times/month	33	33	37	33	30	25
Regular aspirin use (%)	35	46	46	52	49	48
Regular NSAID use (%)	33	40	23	22	N/A	N/A
Family history CRC in parent or sibling (%)	23	17	19	15	N/A	N/A
Total calcium intake (mg/day, mean (SD))	952.4 (364.1)	1007.5 (386.9)	916.3 (399.7)	950.6 (382.5)	N/A	N/A
Total folate intake (μg/day, mean (SD))	426.8 (179.1)	447.8 (194.9)	525.9 (226.3)	566.2 (231.6)	N/A	N/A

N/A: not available.

Within each cohort, 3 controls were randomly selected from participants alive and free of cancer at the time of case ascertainment, and matched to cases based on ethnicity, year of birth and month/year of blood or buccal sampling (NHS and HPFS) [30] and in PHS, controls were additionally matched on smoking status [32].

**Table 2:** Risk of colorectal cancer according to susceptibility SNPs in NHS, HPFS, PHS.

Locus/Gene	rsnumber	Proximity to VDREβ	Tested allele	NHS, HPFS, PHS OR (95% CI)	NHS, HPFS, PHS P-value	Ref	Previous OR (95% CI)	GECCO^1^ OR (95% CI)	GECCO^1^ P-value
11q23/*C11org93*	rs3802842χ	proximalVDRE	A	0.86 (0.78, 0.95)	0.003	2	0.90 (0.87–0.93)	0.90 (0.87–0.94)	4.46E-07
18q21/*SMAD7*	rs4939827χ	proximalVDRE	C	0.92 (0.85, 1.01)	0.076	2,3	0.83 (0.81–0.86)	0.89 (0.86–0.92)	1.66E-10
19q13.1/*RHPN2*	rs10411210χ	proximalVDRE	C	1.10 (0.95, 1.27)	0.209	4	1.15 (1.10–1.20)	1.08 (1.02–1.15)	0.012
8q24/*SRRM1P1/POU5F1B/MYC*	rs6983267χ	nonproxVDRE	G	1.18 (1.08, 1.29)	2.00E-04	5,6,7,8	1.21 (1.18–1.24)	1.13 (1.09–1.18)	1.25E-11
8q23.3/*TRPS1/EIF3H*	rs16892766χ	nonproxVDRE	A	0.89 (0.77, 1.03)	0.109	9	0.80 (0.76–0.84)	0.81 (0.76–0.87)	4.59E-10
10p14/*KRT8P16/TCEB1P3*	rs10795668χ	nonproxVDRE	A	0.94 (0.86, 1.04)	0.221	9	0.89 (0.86–0.91)	0.95 (0.91–0.99)	0.010
14q22.2/*BMP4/MIR5580*	rs4444235χ	nonproxVDRE	C	1.08 (0.99, 1.18)	0.071	10,4	1.09 (1.06–1.12)	1.07 (1.03–1.11)	1.59E-04
15q13/*SCG5/GREM1*	rs4779584χ	nonproxVDRE	C	0.95 (0.85, 1.05)	0.311	10,11	0.87 (0.84–0.91)	0.89 (0.84–0.93)	5.03E-07
16q22.1/*CDH1*	rs9929218χ	nonproxVDRE	A	0.98 (0.89, 1.08)	0.742	4	0.91 (0.89–0.94)	0.95 (0.92–0.99)	0.022
20p12.3/*FERMT1/BMP2*	rs961253χ	nonproxVDRE	A	1.13 (1.04, 1.24)	0.006	10,4	1.12 (1.09–1.15)	1.08 (1.04–1.12)	3.67E-05
1q41/*DUSP10/CICP13*	rs6691170χ	-	G	1.01 (0.92, 1.10)	0.890	12	0.94 (0.92–0.97)	0.98(0.94–1.02)	0.390
1q41/*DUSP10/CICP13*	rs6687758χ	-	A	1.01 (0.92, 1.11)	0.826	12	0.92 (0.89–0.94)	0.95(0.90–1.00)	0.050
3q26.2/*MYNN*	rs10936599χ	-	C	1.02 (0.92, 1.13)	0.726	12	1.08 (1.04–1.10)	0.99(0.94,1.04)	0.630
8q24/*MYC/SRRM1P1/POU5F1B*	rs10505477χ	-	A	1.18 (1.08, 1.29)	2.00E-04	6	1.17(1.12–1.23)	1.14(1.09–1.19)	8.23E-10
8q24/*MYC/SRRM1P1/POU5F1B*	rs7014346χ	-	A	1.19 (1.09, 1.30)	1.00E-04	2	1.19(1.15–1.23)	1.12(1.08–1.17)	4.16E-08
12q13.13/*LARP4/DIP2B*	rs7136702χ	-	C	0.93 (0.85, 1.02)	0.118	12	0.94 (0.93–0.96)	0.91(0.87–0.95)	2.76E-05
12q13.13/*DIP2B/ATF1*	rs11169552χ	-	C	1.08 (0.98, 1.19)	0.128	12	1.09 (1.05–1.11)	1.07(1.02–1.12)	0.004
20q13.33/*LAMA5*	rs4925386α ^,^ χ	-	C	1.02 (0.93, 1.12)	0.700	12	1.08 (1.05–1.10)	1.07(1.02–1.11)	0.005
1q25.3/*LAMC1*	rs10911251δ	-	A	1.04 (0.92, 1.19)	0.522	1	1.09 (1.06–1.13)	1.09 (1.06–1.13)	9.45E-08
2q32.3/*NABP1/SDPR*	rs11903757δ	-	C	1.04 (0.88, 1.25)	0.629	1	1.15 (1.09–1.22)	1.15 (1.08–1.23)	1.38E-06
5q31.1/*PITX1/H2AFY*	rs647161δ	-	A	0.99 (0.85, 1.13)	0.816	13	1.17 (1.11–1.22)	1.07 (1.02–1.11)	0.002
6q21/*SRSF3/CDKN1A*	rs1321311δ	-	A	1.13 (0.98, 1.31)	0.091	14	1.10 (1.07–1.13)	1.04 (1.00–1.08)	0.064
9p24/*TPD52L3/UHRF2/GLDC*	rs719725δ	-	A	1.01 (0.88, 1.15)	0.937	6, 15	1.07 (1.03–1.12)	1.06 (1.02–1.10)	0.001
11q13.4/*POLD3*	rs3824999δ	-	G	1.14 (1.00, 1.29)	0.049	14	1.08 (1.05–1.10)	1.08 (1.04–1.12)	3.43E-05
12p13.32/*CCND2*	rs3217810δ	-	T	1.20 (0.94, 1.52)	0.138	1	1.20 (1.12–1.28)	1.20 (1.12–1.28)	5.86E-08
12p13.32/*CCND2*	rs3217901δ	-	G	1.06 (0.94, 1.21)	0.339	1	1.10 (1.06–1.14)	1.10 (1.06–1.14)	3.31E-07
12p13.32/*RPL18P9/CCND2*	rs10774214δ	-	T	0.93 (0.80, 1.09)	0.394	3	1.04 (1.00–1.09)	1.04 (0.99, 1.08)	0.120
12q24.21/*TBX3*	rs59336δ	-	T	1.05 (0.91, 1.20)	0.508	1	1.09 (1.06–1.13)	1.09 (1.06–1.13)	3.67E-07
14q22.2/*BMP4/ATP5C1P1/CDKN3*	rs1957636δ	-	C	1.08 (0.95, 1.23)	0.253	10	0.92 (0.90–0.95)	0.95 (0.92–0.99)	0.006
15q13/*SCG5/GREM1*	rs11632715δ	-	A	1.02 (0.90, 1.16)	0.709	10	1.12 (1.08–1.16)	1.05 (1.01–1.09)	0.012
15q13/*SCG5/GREM1*	rs16969681δ	-	C	0.87 (0.70, 1.10)	0.244	10	0.84 (0.80–0.90)	0.93 (0.87–0.99)	0.018
20p12.3/*BMP2/HAO1*	rs2423279δ	-	C	1.11 (0.96, 1.28)	0.167	13	1.07 (1.03–1.12)	1.06 (1.01, 1.11)	0.010
20p12.3/*FERMT1/BMP2*	rs4813802δ	-	G	0.99 (0.87, 1.13)	0.890	10, 16	1.09 (1.06–1.12)	1.10 (1.05–1.14)	6.99E-06

αWe used a surrogate rs2151512 for rs4925386 (linkage disequilibrium r^2^ 1.0 in the HapMap CEU population).

βProximity to vitamin D response element (VDRE) based on published ChIP-seq data[26].

χSNPs genotyped with TaqMan among 1895 CRC cases and 2806 controls.

δSNPs genotyped and imputed off Illumina HumanOmniExpress among 954 CRC cases and 1328 controls.

References: 1. Peters et al. Gastroenterology 2012; 2. Tenesa et al. Nature Genetics 2008; 3. Broderick et al. Nature Genetics 2007; 4. COGENT Nature Genetics 2008; 5. Tomlinson et al. Nature Genetics 2007; 6. Zanke et al. Nature Genetics 2007; 7. Haiman et al. Nature Genetics 2007; 8. Hutter et al. BMC Cancer 2010; 9. Tomlinson et al. Nature Genetics 2008; 10. Tomlinson et al. PLoS Genet, 2011; 11. Jaeger et al. Nature Genetics 2008; 12. Houlston et al. Nature Genetics 2010; 13. Jia et al. Nat Genet, 2012. 14. Dunlop et al. Nat Genet, 2012. 15. Kocarnik et al. CEBP, 2010. 16. Peters et al. Hum Genet, 2011.

The additive GRSs comprised of the CRC risk alleles yielded effect estimates for CRC within our three cohorts that were comparable with those derived from all 13 cohorts encompassed by GECCO and CCFR (Table 3). We also compared the estimates obtained from models with the GRS-proximalVDRE and GRS-nonproximalVDRE scores alone with models containing both GRSs (conditional models) and did not observe a material difference. There was no significant difference between a GRS-VDR (multivariate OR, 1.12; 95% CI 1.06, 1.19), and a GRS-nonproximalVDRE (multivariate OR, 1.10; 95% CI 1.06, 1.14) (p-heterogeneity  =  0.52).

**Table 3:** Comparison of GRS-31, GRS-18, GRS-10, GRS-proximalVDR and GRS-nonproxVDR.

	n (cases/controls)	GRSproximalVDRα OR (95% CI)	GRSnonproxVDRβ OR (95% CI)	GRS-10χ OR (95% CI)	GRS-18δ OR (95% CI)	GRS-31ε OR (95% CI)
**NHS**	2358 (922/1436)	1.10 (1.01, 1.20)	1.13 (1.07, 1.19)	1.12 (1.07, 1.17)	1.07 (1.04, 1.10)	1.05 (1.02, 1.09)
**HPFS**	1457 (586/871)	1.13 (1.01, 1.26)	1.08 (1.01, 1.15)	1.09(1.03, 1.16)	1.05 (1.01–1.09)	1.04 (0.98, 1.10)
**PHS**	886 (387/499)	1.15 (1.01, 1.31)	1.07 (0.98, 1.17)	1.09 (1.01, 1.18)	1.07 (1.02–1.12)	1.05 (1.00, 1.10)
**Meta analysis**	4701 (1895/2806)	1.12 (1.06, 1.19)	1.10 (1.06, 1.14)	1.10 (1.07, 1.14)	1.06 (1.04–1.09)	1.05 (1.02, 1.08)
**GECCO**	22829 (10061/12768)	1.11 (1.09, 1.14)	1.09 (1.07, 1.11)	1.10 (1.08, 1.11)	1.08 (1.07, 1.09)	1.08 (1.07, 1.09)

αGRS-proximalVDR: additive genetic risk score used to estimate a per allele OR and 95% CI. Includes SNPs; rs3802842, rs4939827, rs10411210.

βGRS-nonproxVDR: additive genetic risk score used to estimate a per allele OR and 95% CI. Includes SNPs; rs6983267, rs16892766, rs10795668, rs4444235, rs4779584, rs9929218, rs961253.

χGRS-10: additive genetic risk score used to estimate a per allele OR and 95% CI. Includes SNPs; rs3802842, rs4939827, rs10411210, rs6983267, rs16892766, rs10795668, rs4444235, rs4779584, rs9929218, rs961253.

δGRS-18: additive genetic risk score used to estimate a per allele OR and 95% CI. Includes SNPs; rs3802842, rs4939827, rs10411210, rs6983267, rs16892766, rs10795668, rs4444235, rs4779584, rs9929218, rs961253, rs6691170, rs6687758, rs10936599, rs10505477, rs7014346, rs7136702, rs11169552, rs2151512.

εGRS-31: additive genetic risk score used to estimate a per allele OR and 95% CI based upon the subset of cases and controls with GWAS data (394 cases/772 controls in NHS; 228 cases/222 controls in HPFS; 332 cases/332 controls in PHS). Includes SNPs; rs3802842, rs4939827, rs10411210, rs6983267, rs16892766, rs10795668, rs4444235, rs4779584, rs9929218, rs961253, rs6691170, rs6687758, rs10936599, rs7136702, rs11169552, rs4925386, rs10911251, rs11903757, rs647161, rs1321311, rs719725, rs3824999, rs3217810, rs3217901, rs10774214, rs59336, rs1957636, rs11632715, rs16969681, rs2423279, rs4813802.

Consistent with our prior reports [23], [32], we observed an inverse association between 25(OH)D and CRC in a meta-analysis of results from the three cohorts (Table 4). Compared with individuals in the lowest quartile of 25(OH)D, men and women in the highest quartile had a multivariate OR for CRC of 0.63 (95% CI 0.48–0.82; p-trend<0.001), and for those with higher levels of 25(OH)D (≥ 32 ng/mL) compared with lower levels, the multivariate OR was 0.79 (95% CI 0.65–0.98; p = 0.03).

**Table 4:** Risk of colorectal cancer associated with circulating 25(OH)D within NHS, HPFS and PHS.

	NHS	HPFS	PHS	Meta-analysis
N (case/control)	352/665	277/535	252/366	881/1566
Continuous 25(OH)D	0.98 (0.97, 1.00)	0.99 (0.97, 1.01)	1.01 (0.99, 1.03)	0.99 (0.98, 1.00)
High vitamin D (≥ 32 ng/ml)	0.76 (0.54, 1.08)	0.77 (0.56, 1.05)	0.91 (0.59, 1.41)	0.79 (0.65, 0.98)
Quartile 1	1.00 (referent)	1.00 (referent)	1.00 (referent)	1.00 (referent)
Quartile 2	0.77 (0.53, 1.12)	0.90 (0.60, 1.35)	0.70 (0.41, 1.19)	0.80 (0.62, 1.02)
Quartile 3	0.58 (0.39, 0.87)	0.64 (0.41, 0.99)	0.91 (0.54, 1.52)	0.67 (0.52, 0.86)
Quartile 4	0.57 (0.38, 0.87)	0.67 (0.42, 1.05)	0.67 (0.39, 1.17)	0.63 (0.48, 0.82)

**Figure 1:**
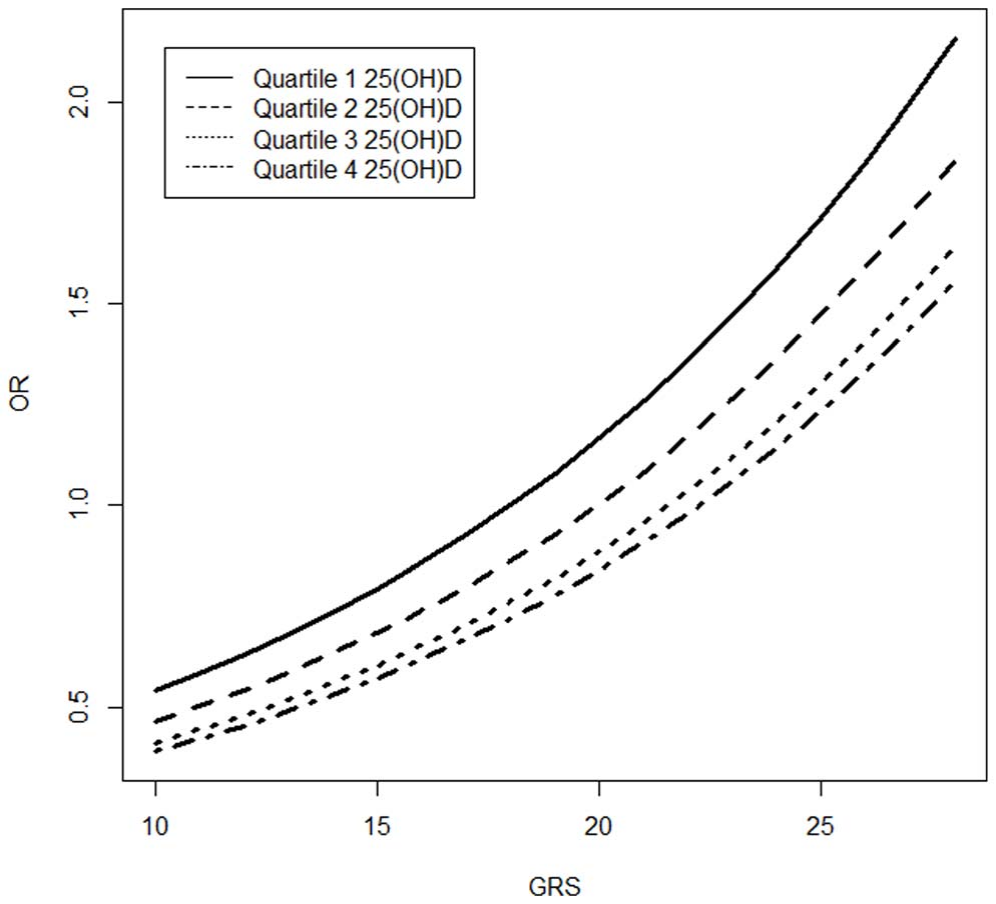
GRS-31 and CRC stratified by quartiles of 25(OH)D. Demonstrated no evidence for variation in CRC risk estimates associated with GRS-31, stratified across quartiles of 25(OH)D (phet = 0.98).

Tests of multiplicative interactions between continuous GRSs and continuous 25(OH)D did not yield statistically significant results, nor did tests of multiplicative interactions between GRSs and vitamin D categorized by a threshold level of 32 ng/mL. Likewise, the risk estimates associated with GRS31 did not vary according to quartile of vitamin D (phet = 0.98) (Figure 1).

## Discussion

There is substantial evidence supporting an inverse association between circulating 25(OH)D and CRC risk; meta-analyses and systematic reviews have observed a 50% lower risk of CRC comparing extreme quintiles of 25(OH)D [21], [24]. Several mechanisms have been hypothesized to underlie this association, some of which may be shared by pathways associated with the putative functional consequences of CRC susceptibility SNPs proximal to VDR-DNA binding sites. In addition, vitamin D signaling occurs through binding of the active form 1,25(OH)D to nuclear vitamin D receptor (VDR) along specific genomic sequences known as vitamin D response elements (VDREs),) which act to activate or repress gene transcription [25]. In our study of 1895 CRC cases and 2806 controls nested within three prospective cohorts, we did not observe statistically significant evidence for interaction between CRC genetic markers, including the 3 CRC SNPs identified in a prior CHiP-seq analysis as being adjacent to VDR-DNA binding sites, and plasma 25(OH)D on CRC risk, despite observing an increased risk for CRC associated with GRSs and an inverse association between circulating 25(OH)D levels and CRC. [26].

The lack of a significant interaction between 25(OH)D and genetic susceptibility to CRC may have several explanations. First, pathways associated with CRC susceptibility loci may in fact be distinct or overlap minimally with mechanisms associated with vitamin D. Second, our GRS and single measures of plasma 25(OH)D measures may be relatively insensitive or incomplete markers of relevant biological pathways shared by genetic susceptibility and vitamin D. Third, we constructed GRSs informed by the results of a functional study by Ramagopalan et al. that applied ChiP-seq in lymphoblastoid cells treated with calcitriol for 36 hours to demonstrate subsequent differential association of vitamin D receptor binding with three specific CRC susceptibility loci [26]. However, a separate study utilizing ChIP-Seq in monocytes treated with calcitriol for 40 minutes found only 18% of calcitriol-stimulated VDR-binding sites common to those observed in Ramagopalan et al. [37], [38]. These results suggest that VDR target gene regulation may differ according to cell type and/or duration of vitamin D exposure which may be difficult to assess using circulating measures of 25(OH)D.

Our study has several strengths. First, the availability of both genetic information and prediagnostic measures of plasma 25(OH)D in our three cohorts permitted, to our knowledge, the first analysis of the effect of CRC susceptibility loci according to an integrated biomarker of an environmental determinant of CRC risk. Second, our reasonably large sample size provided individual SNP and GRS associations that were similar in the direction and magnitude with estimates from larger cohorts, including the GECCO and CCFR consortium. We acknowledge limitations of our study, including a single measurement of 25(OH)D which may not reflect long-term vitamin D status or the tissue-specific effects of vitamin D. We also had a more limited sample size of participants with both genetic information and measured levels of plasma 25(OH)D.

In summary, in this large study of CRC cases and controls characterized for genetic susceptibility to CRC with prediagnostic measurements of 25(OH)D levels, we did not observe evidence for the modification of genetic susceptibility for CRC according to vitamin D status.
